# Deep learning radio-clinical signatures for predicting neoadjuvant chemotherapy response and prognosis from pretreatment CT images of locally advanced gastric cancer patients

**DOI:** 10.1097/JS9.0000000000000432

**Published:** 2023-05-03

**Authors:** Can Hu, Wujie Chen, Feng Li, Yanqiang Zhang, Pengfei Yu, Litao Yang, Ling Huang, Jiancheng Sun, Shangqi Chen, Chengwei Shi, Yuanshui Sun, Zaisheng Ye, Li Yuan, Jiahui Chen, Qin Wei, Jingli Xu, Handong Xu, Yahan Tong, Zhehan Bao, Chencui Huang, Yiming Li, Yian Du, Zhiyuan Xu, Xiangdong Cheng

**Affiliations:** a Department of Gastric Surgery; b Department of Radiology, The Cancer Hospital of the University of Chinese Academy of Sciences (Zhejiang Cancer Hospital), Institutes of Basic Medicine and Cancer (IBMC), Chinese Academy of Sciences, Hangzhou; c Department of Research Collaboration, R&D Center, Beijing Deepwise & League of PHD Technology Co., Ltd, Beijing; d Department of Gastrointestinal Surgery, The Affiliated Hospital of Wenzhou Medical University, Wenzhou; e Department of General Surgery, HwaMei Hospital, University of Chinese Academy of Sciences, Ningbo; f Department of Gastrointestinal Surgery, The Affiliated Hospital of Zhejiang Chinese Medical University; g Department of Gastrointestinal Surgery, Tongde Hospital of Zhejiang Province; h Department of Gastric Surgery, Fujian Cancer Hospital; i Key Laboratory of Prevention, Diagnosis and Therapy of Upper Gastrointestinal Cancer of Zhejiang Province; j Zhejiang Provincial Research Center for Upper Gastrointestinal Tract Cancer, Zhejiang Cancer Hospital, Hangzhou, People’s Republic of China

**Keywords:** deep learning, locally advanced gastric cancer, neoadjuvant chemotherapy, prognosis, tumor regression grade

## Abstract

Early noninvasive screening of patients who would benefit from neoadjuvant chemotherapy (NCT) is essential for personalized treatment of locally advanced gastric cancer (LAGC). The aim of this study was to identify radio-clinical signatures from pretreatment oversampled computed tomography (CT) images to predict the response to NCT and prognosis of LAGC patients.

**Methods:**

LAGC patients were retrospectively recruited from six hospitals from January 2008 to December 2021. An SE-ResNet50-based chemotherapy response prediction system was developed from pretreatment CT images preprocessed with an imaging oversampling method (i.e. DeepSMOTE). Then, the deep learning (DL) signature and clinic-based features were fed into the deep learning radio-clinical signature (DLCS). The predictive performance of the model was evaluated based on discrimination, calibration, and clinical usefulness. An additional model was built to predict overall survival (OS) and explore the survival benefit of the proposed DL signature and clinicopathological characteristics.

**Results:**

A total of 1060 LAGC patients were recruited from six hospitals; the training cohort (TC) and internal validation cohort (IVC) patients were randomly selected from center I. An external validation cohort (EVC) of 265 patients from five other centers was also included. The DLCS exhibited excellent performance in predicting the response to NCT in the IVC [area under the curve (AUC), 0.86] and EVC (AUC, 0.82), with good calibration in all cohorts (*P*>0.05). Moreover, the DLCS model outperformed the clinical model (*P*<0.05). Additionally, we found that the DL signature could serve as an independent factor for prognosis [hazard ratio (HR), 0.828, *P*=0.004]. The concordance index (C-index), integrated area under the time-dependent ROC curve (iAUC), and integrated Brier score (IBS) for the OS model were 0.64, 1.24, and 0.71 in the test set.

**Conclusion:**

The authors proposed a DLCS model that combined imaging features with clinical risk factors to accurately predict tumor response and identify the risk of OS in LAGC patients prior to NCT, which can then be used to guide personalized treatment plans with the help of computerized tumor-level characterization.

## Introduction

Highlights

- We developed and validated deep learning radio-clinical signatures (DLCS) from pretreatment CT images to predict the preoperative chemotherapy response and prognosis in locally advanced gastric cancer (LAGC) patients.
- The proposed DLCS has promising performance in predicting preoperative chemotherapy response and prognosis.
- The DLCS model may guide treatment plans and implementation of personalized treatment for LAGC patients treated with preoperative chemotherapy.

Gastric cancer (GC) is among the most prevalent gastrointestinal malignancies globally and is the third leading cause of cancer-related deaths worldwide^1^. While surgery remains the primary treatment for GC, radical gastrectomy is only suitable for patients diagnosed with early GC. Unfortunately, 50–60% of patients are typically diagnosed in advanced stages, with invasion or metastasis^2^. After radical surgery, patients with locally advanced gastric cancer (LAGC), which refers to the stage between early GC and advanced GC, face high rates of distant metastasis and local recurrence, ranging from 40 to 51%^3^.

In recent years, neoadjuvant chemotherapy (NCT) has been shown to significantly improve the prognosis of patients with LAGC and has become the standard treatment^4^. NCT improves overall survival (OS) and disease-free survival (DFS) mainly by reducing the tumor volume, achieving tumor degradation, and eliminating micrometastases as early as possible to increase the probability of R0 resection through chemotherapy before surgery. Moreover, maintaining tumor vascular integrity prior to surgery can enhance the efficacy of chemotherapy. However, not all LAGC patients can benefit from NCT, and ineffective neoadjuvant therapy may increase toxicity and allow tumors to progress during chemotherapy^5,6^. Therefore, early screening is essential to identify patients who are likely to benefit from NCT upon diagnosis.

Currently, the tumor regression grade (TRG) is considered the gold standard for evaluating the effectiveness of NCT. TRG is determined by examining postoperative tissue specimens under a microscope and assessing the degree of tumor regression^7^. However, this standard relies on the acquisition of complete specimens after surgery, which limits its use in preoperative clinical practice. The development of a noninvasive method for accurately identifying patients who are responsive to NCT holds significant clinical value. With the development of technology, radiomics has rapidly developed as a new tool for noninvasive tumor analysis^8,9^. Pretreatment imaging is linked to primary tumor characteristics, whereas posttreatment images can directly indicate the response of the tumor to chemotherapy. Some studies have confirmed that some radiomic features are significantly associated with the chemotherapy response and can be used to create a radiomics-based model for predicting the NCT response in patients with cancer^10,11^. However, there are some limitations to radiomic feature extraction, and it can be prone to producing deviations. In recent years, deep learning (DL) signatures based on convolutional neural networks^12^ have been shown to better reveal the biological information reflected by computed tomography (CT) images. However, few studies have developed DL signatures to preoperatively predict the pathological response in GC patients. The pretrained ResNet architecture has been widely used to assist in the diagnosis of different kinds of diseases via transfer learning^13–15^, especially with the added block of SE-Net^16,17^. To the best of our knowledge, no imaging oversampling method has been applied in this area to balance data.

Therefore, the objective of this study was to develop and validate a deep learning radio-clinical signature (DLCS) model, utilizing an imaging sampler, for early prediction of the response to chemotherapy before administering NCT in a large multicenter patient cohort. Furthermore, we investigated the added value of the DL signature in predicting OS in the follow-up cohort.

## Patients and methods

### Patients

We retrospectively analyzed the data of consecutive patients with histologically confirmed GC/esophagogastric junction cancer (EGJC) who received NCT with a 5-fluorouracil-based regimen prior to surgical resection at six independent hospitals in China. This trial was registered on the ClinicalTrials network (http://www.clinicaltrial.gov) under the identifier NCT05617469. The work is reported in line with the STROCSS (Strengthening The Reporting of Cohort Studies in Surgery) criteria^18^, Supplemental Digital Content 1, http://links.lww.com/JS9/A433. The inclusion criteria were as follows: patients with GC or Siewert type III EGJC confirmed by pathological examination; patients diagnosed locally advanced stage (cT1N+, cT2-4N0/+M0, partial M1 patients underwent conversion therapy); patients who underwent gastrectomy plus lymphadenectomy; patients who received at least two cycles of preoperative chemotherapy; patients with negative resection margins; patients with complete CT image data and clinical data. The exclusion criteria were as follows: patients unable to undergo gastrectomy after neoadjuvant therapy; patients with incomplete CT images and clinical data; patients with other malignancies.

The medical records of all patients were reviewed. Baseline clinicopathological data, including age, sex, BMI, tumor location, maximum diameter, Borrmann type, level of blood parameters before NCT, and differentiation degree, were collected. According to the 8th American Joint Committee on Cancer TNM (tumor-node-metastasis) staging system, cT (clinical tumor) stage, cN (clinical nodal) stage, and cM (clinical metastasis) stage were also retrieved from medical records. The study was approved by the ethics committees of all participating centers (IRB-2022-371). The study is consistent with the tenets of the Declaration of Helsinki. Since this study was retrospective, the requirement to obtain informed consent from the patients was waived.

After two to three cycles of NCT, clinical efficacy was assessed based on CT or magnetic resonance imaging (MRI). After systemic treatment, the patients were regularly examined every 3 months for the first year and every 6 months thereafter. The final follow-up assessment was conducted in December 2021.

### NCT protocols

All patients received a minimum of two cycles of NCT with a 5-fluorouracil-based regimen according to the guidelines for the treatment of GC before undergoing gastrectomy with lymphadenectomy. Gastrectomy and lymph node dissection were performed within 2 weeks after completing NCT.

### Pathological response assessment

All patients underwent gastrectomy after completing NCT, and the resected specimens were evaluated by two experienced pathologists who were blinded to the clinical and imaging data of the patients. Pathological TRG was used to assess the pathological response. TRG scores were evaluated using the American Society of Clinical Oncology/College of American Pathologists criteria, which were included in the third edition of the National Comprehensive Cancer Network Guidelines for Gastric Cancer in 2017 and are routinely recommended by the Chinese Society of Clinical Oncology. The four levels were as follows: the absence of residual cancer cells was defined as TRG 0; the presence of single cells or small groups of cells was defined as TRG 1; the presence of residual carcinoma with connective tissue hyperplasia was defined as TRG 2; and minimal evidence of tumor response was defined as TRG 3. The patients were divided into the good response (GR) group, which included TRG 0 and TRG 1, and the poor response (PR) group, which included TRG 2 and TRG 3.

### CT examination and ROI delineation

All patients underwent an enhanced CT examination within 1 week prior to starting NCT. Tumor segmentation was performed by two experienced radiologists via ITK-SNAP software (version 3.8, http://www.itksnap.org). Since GC can be distinguished from normal gastric tissues in portal venous phase CT images, three slices, including the two-dimensional (2D) slice with the largest tumor and its two nearest slices in the *z*-axis, were delineated along the boundaries of the tumor in portal venous phase CT images. When there was a large dispute between the two radiologists on the region of interest (ROI) delineation, the two radiologists reached a consensus after discussion.

### Data acquisition

The 2D slice with the largest tumor and its two nearest slices on the *z*-axis were used as input data per patient. All the images were first normalized to a size of 1.0×1.0 mm^2^ and filtered with a window of [−115, 235] HU. Then, the input images, which had a size of 112×112 and focused on the manually delineated tumor section with an expansion of 5 mm in all directions, were included in the subsequent analysis. The pixel values of the image were normalized to [0, 1]. During training, DeepSMOTE^19^, a novel image sampler, was applied to balance the dataset at a 1:1 ratio (GR cases:PR cases) in an oversampling way, which enables rich information about minority classes and reduces blurred class boundaries. Flipping and rotation were employed as data augmentation strategies before the images were fed into the network in the training set.

### Development of TRG signatures

**Figure 1:**
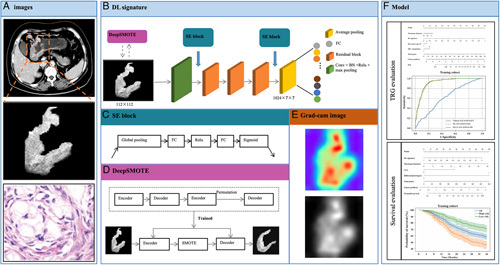
Workflow in this study. Workflow of developing deep learning signatures to building models for predicting neoadjuvant chemotherapy response and prognosis in LAGC patients. BN, batch normalization; DL, deep learning; FC, fully connection; LAGC, locally advanced gastric cancer; SE, squeeze and excitation; TRG, tumor regression grade.

The workflow for building TRG signatures is shown in Figure 1. To develop a DL signature for predicting TRG classification, we designed the pretrained Resnet50 on ImageNet to have only four stages, which consisted of three, four, and six residual blocks. Inspired by the idea of SE-Net (Function S1, Supplemental Digital Content 2, http://links.lww.com/JS9/A434)^17^, which adopts two consecutive processes, including squeeze and excitation, to capture the implicit interdependency of channels, we added a channel-attention block before the first and after the last residual blocks to improve the feature representations generated by the network. We changed softmax to sigmoid as the final layer to produce the probabilistic predictions of a binary classifier. For the end-to-end classification model, the size of the final feature map outputs was 7×7, which was the size of the original input image downsampled four times. The DL signature was fine-tuned on the training set using five-fold cross-validation. For the training stage, the model was developed with a mini-batch size of 32, and the learning rate was initially set to 0.0005 with a decay rate of 0.1 every 50 epochs. For the reasoning stage, we used the Gradient-weighted Class Activation Map (Grad-CAM)^20^ to visualize the suspicious tumor area detected by the network for making decisions regarding GR and PR. The DL signature was trained on two GeForce RTX 2080 Ti GPUs with the PyTorch framework for 2000 epochs at maximum, and the early-stopping function was set to 100 consecutive epochs.

A total of 22 clinicopathologic characteristics were analyzed for clinical TRG classification model (clinical signature) construction using a multivariate logistic regression (MLR)^21^ algorithm in the training cohort (TC). The pairwise correlations of included factors were determined by Spearman’s *ρ*. Least absolute shrinkage and selection operator^22^ was then applied to select the most valuable sparse feature matrix. Subsequently, a fusion signature for the DLCS was built from the significant image-based and clinic-based features (univariable analysis, *P*<0.05) using MLR.

### Association between the DL signature and prognosis

To explore the additional value of the proposed image-based DL signature, in combination with significant clinicopathological characteristics, for predicting OS, we first used univariate Cox regression analysis^23^ to screen for independent risk factors. The selected features were then integrated to develop a prognostic model using the multivariate Cox regression (MCR) method^24^ in the training set.

The training and test sets for OS prediction were randomly split from the follow-up data of 654 LAGC patients at a ratio of 8:2. A nomogram for pretherapy OS prediction was built and evaluated on this new dataset.

### Statistical analysis

The DL signature was implemented using PyTorch (version 1.7.1), and all statistical analyses were conducted in Python 3.8. The Mann–Whitney *U* test was applied for data with a nonnormal distribution. Student’s *t*-test and the chi-square ( $\chi^{2}$ ) test were used for continuous and categorical data, respectively, with a normal distribution. For TRG classification models, receiver operating characteristic (ROC) analysis and precision-recall curves were performed using the continuous probability score (range: [0, 1]). Decision curve analysis (DCA) was used to evaluate the clinical usefulness of the TRG prediction models by quantifying the net benefit at various threshold probabilities. Calibration curves and smoothed calibration curves were used for the classification model and survival probability calibration, respectively. For survival models, the integrated area under the time-dependent ROC curve (iAUC)^25^ was calculated, the discriminatory capacity was evaluated using the concordance index (C-index), and the error was assessed by the integrated Brier score (IBS)^26^. In addition, the log-rank test with Kaplan–Meier survival curves^27^ was used to verify the model’s discriminatory ability. A *P* value <0.05 was used to indicate a statistically significant difference.

## Results

### Baseline information

In the end, 1060 patients were included in this study. A total of 664 patients who received NCT prior to surgical resection at center I from January 2008 to December 2019 were enrolled as the TC. A total of 131 patients at center I from January 2020 to December 2021 were enrolled as the internal validation cohort (IVC). In addition, 265 patients were enrolled from five independent centers from January 2014 to December 2021 as an external validation cohort (EVC). Among 664 LAGC patients at center I, 654 patients were followed up after discharge from hospital, while 10 patients were never followed up. The workflow of the cohorts is shown in Figure S1, Supplemental Digital Content 2, http://links.lww.com/JS9/A434.

As shown in Table 1, the GR rates in the training, internal validation, and EVCs were 24.40%, 22.14%, and 22.26%, respectively. There was no significant difference in age, sex, BMI, cM stage, or differentiation degree before starting NCT between the GR group and PR group in the three cohorts. In addition, maximum tumor diameter and cT stage showed a significant difference between the GR group and PR group in the TC. Tumor location showed significant differences between the GR group and PR group in the IVC, while there were significant differences in maximum tumor diameter, Borrmann type, cT stage and cN stage between the GR group and PR group in the EVC.

**Table 1:** Clinicopathological characteristics of patients with LAGC in the training and validation cohorts

	Training cohort (664)			Internal validation cohort (131)			External validation cohort (265)		
Characteristics	GR	PR	*P*	GR	PR	*P*	GR	PR	*P*
Age (year)									
≥60	90	283	0.855	16	61	0.655	39	134	0.881
<60	72	219		13	41		20	72	
Sex									
Female	42	132	0.926	10	26	0.338	13	52	0.614
Male	120	370		19	76		46	154	
BMI (kg/m^2^)									
<18.5	21	44	0.068	3	11	0.424	6	20	0.920
18.5–23.9	115	338		22	66		37	135	
≥24.0	25	113		4	25		16	51	
Location									
Upper 1/3	50	149	0.970	6	37	0.011^*^	13	33	0.551
Middle1/3	37	113		14	25		8	42	
Lower 1/3	62	194		9	29		24	84	
Whole stomach	13	46		0	11		14	47	
Maximum diameter									
>5 cm	93	359	<0.001^*^	22	83	0.511	22	118	0.007^*^
≤5 cm	69	139		7	19		37	88	
Borrmann type									
I+II	71	220	1.000	11	42	0.753	16	22	0.001^*^
III+IV	91	282		18	60		43	184	
cT stage									
T1+T2	20	26	0.002^*^	5	12	0.645	10	2	<0.001^*^
T3+T4	142	476		24	90		49	204	
cN stage									
N0+N1	38	100	0.335	7	28	0.722	16	24	0.003^*^
N2+N3	124	402		22	74		43	182	
cM stage									
M0	140	413	0.218	26	88	0.869	59	195	0.149
M1	22	89		3	14		0	11	
Differentiated degree									
Poorly/poorly–middle	127	412	0.298	23	89	0.439	45	160	0.821
Middle/well	35	90		6	13		14	46	
Pathological type									
Adenocarcinoma	161	494	0.587	28	99	1.000	55	203	0.074
Others	1	8		1	3		4	3	
CEA									
Positive	39	152	0.100	10	40	0.173	23	73	0.545
Negative	121	336		16	60		36	131	
Unknown	2	14		3	2		0	2	
CA125									
Positive	14	52	0.448	3	23	0.075	8	31	0.574
Negative	147	443		25	79		51	173	
Unknown	1	7		1	0		0	2	
AFP									
Positive	15	42	0.246	2	9	0.055	10	19	0.042
Negative	144	446		23	91		49	179	
Unknown	3	14		4	2		0	8	
ALB	39.50±4.32	40.07±4.29	0.141	39.99±4.63	39.84±4.48	0.875	38.66±6.27	38.64±5.54	0.983
PCT	0.25±0.08	0.24±0.08	0.549	0.26±0.06	0.25±0.09	0.427	0.29±0.09	0.25±0.08	0.002^*^
Lymph%	27.88±8.68	27.43±8.70	0.575	27.88±9.51	25.99±9.96	0.363	24.12±10.03	25.70±9.70	0.339
Glu	5.46±1.24	5.62±1.39	0.186	5.60±1.34	5.74±1.65	0.681	6.26±1.77	6.54±12.62	0.887
Neut	4.08±1.90	4.04±1.96	0.831	3.90±1.57	4.07±2.00	0.672	4.44±1.75	4.06±1.81	0.225

* *P*<0.05.

Adenocarcinoma group includes adenocarcinoma, mucinous adenocarcinoma, and signet ring cell carcinoma; Others group includes squamous carcinoma and other carcinomas.

AFP, alpha-fetoprotein; ALB, albumin; BMI, body mass index; CA125, cancer antigen 125; CEA, carcinoembryonic antigen; cM, clinical metastasis; cN, clinical nodal; Glu, glucose; GR, good response; LAGC, locally advanced gastric cancer; PCT, platelet crit; PR, poor response.

### Diagnostic performance of the TRG signatures

**Figure 2:**
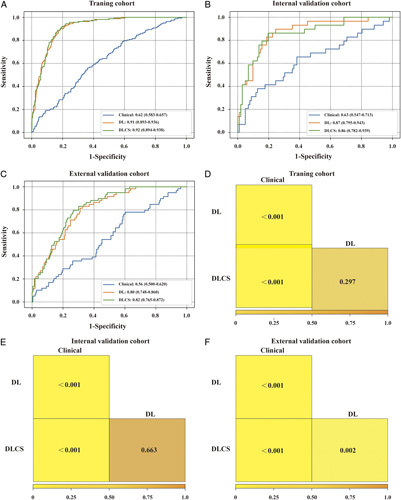
The performance of the three models. (A–C) Receiver operating characteristic (ROC) curves of the clinical model, DL signature, and DLCS model for predicting neoadjuvant chemotherapy response in the training cohort, internal cohort, and external cohort. (D–F) Comparison of the performance of the three models in training cohort, internal cohort, and external cohort. DL, deep learning; DLCS, deep learning radio-clinical signature.

**Figure 3:**
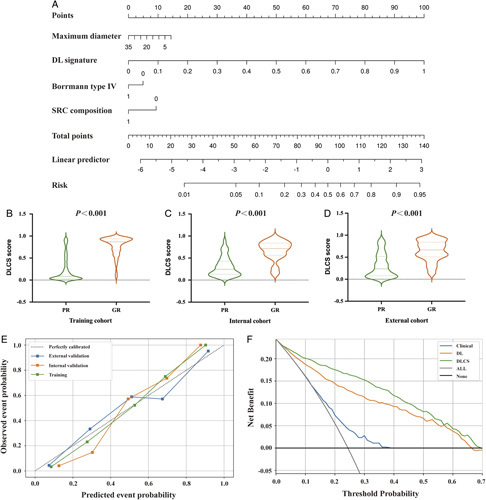
Construction and performance of deep learning radio-clinical signature (DLCS) model. (A) A fusion signature of DLCS was built from the significant DL signature and clinic-based features, including the maximum diameter of the tumor, Borrmann type, and SRC composition. (B–D) The relationship between DLCS score and NCT response in the training cohort, internal cohort, and external cohort. (E) Calibration curves of DLCS model in all three cohorts. (F) Decision curve analysis for the clinical model, DL signature, and DLCS model. DL, deep learning; DLCS, deep learning radio-clinical signature; GR, good response; NCT, neoadjuvant chemotherapy; PR, poor response; SRC, signet ring cell.

Based on internal five-fold cross-validation in the training set, three TRG signatures with the best area under the ROC curves (AUCs) in the validation set were obtained (Fig. 2A). Their corresponding performance outcomes and comparisons in the independent internal and external validation sets are summarized in Table 2 (Fig. 2B, C). The PR curves have been shown in Figure S2, Supplemental Digital Content 2, http://links.lww.com/JS9/A434. The DL signature had a better discriminatory ability than the clinical signature (*P*<0.0001), with AUCs of 0.91 (95% CI, 0.893–0.936) and 0.62 (95% CI, 0.583–0.657), respectively. With comparable performance outcomes, the DLCS had slightly higher AUC, accuracy, and specificity values than the DL signature except for the slightly lower sensitivity, with values of 0.92 vs. 0.91 (*P*=0.297, DeLong test), 0.84 vs. 0.82, 0.82 vs. 0.80, and 0.89 vs. 0.91, respectively. Moreover, nearly the same comparable outcomes of all models were validated in the independent IVC and EVC. Although the DLCS achieved only slightly higher values than the DL signature, it showed consistently better outcomes in all datasets, especially for accuracy and specificity. The DL signature was further confirmed its good performance to predict chemotherapy response in all subgroups in the independent internal and external validation sets (Figure S3A, B, Supplemental Digital Content 2, http://links.lww.com/JS9/A434). Besides, it has been verified as an independent risk factor in the subgroups for OS prediction (Figure S3C, Supplemental Digital Content 2, http://links.lww.com/JS9/A434). In conclusion, the results revealed that the DLCS with complementary multimodality information has a better and more robust TRG diagnostic ability than any single-source model (Table 2 and Fig. 2D–F). The nomogram based on the DLCS is displayed in Figure 3A. There was a significant difference in the DLCS score between the GR group and PR group in the three cohorts (Fig. 3B–D). We observed that the DLCS was well calibrated in all cohorts and had a larger net benefit than the other signatures in the whole dataset (Fig. 3E, F).

**Table 2:** The accuracy, sensitivity, and specificity of different models in all data sets

Cohorts	Models	Accuracy	Sensitivity	Specificity
Training cohort	DL signature	0.82	0.91	0.80
	Clinical model	0.56	0.64	0.54
	DLCS model	0.84	0.89	0.82
Internal cohort	DL signature	0.78	0.90	0.75
	Clinical model	0.66	0.52	0.69
	DLCS model	0.82	0.86	0.80
External cohort	DL signature	0.72	0.78	0.70
	Clinical model	0.52	0.54	0.51
	DLCS model	0.74	0.76	0.74

DL signature, deep learning signature; DLCS model, deep learning radio-clinical signature model.

Furthermore, we analyzed the relationship between the DL signature and clinicopathologic characteristics (as shown in Figure S4, Supplemental Digital Content 2, http://links.lww.com/JS9/A434). We found that the DL signature were significantly correlated with signet ring cell (SRC) composition (*P*=0.021), cT stage (*P*=0.002), and maximum diameter of the primary tumor (*P*<0.001), while there was no correlation between the DL signature and sex, age, BMI, differentiation degree, number of extra-lymph node metastases (ELNs), cN stage, cM stage, Borrmann type, location, pathological type, or some blood parameters, including carcinoembryonic antigen (CEA) level, cancer antigen 125 (CA125) level, alpha-fetoprotein (AFP) level, neutrophil level, lymph% level, platelet crit (PCT) level, albumin (ALB) level, and glucose (Glu) level (*P*>0.001).

**Figure 4:**
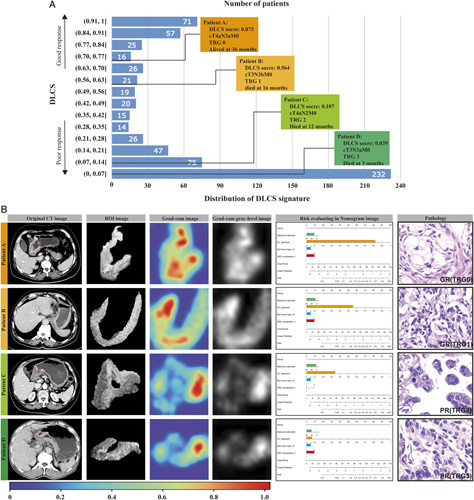
Distribution of DLCS scores in the training cohort (A) and some representative examples of computed tomography images and visualization of SE-Resnet50 prediction using guided Gram (B). CT, computed tomography; DLCS, deep learning radio-clinical signature; GR, good response; PR, poor response; TRG, tumor regression grade.

The Grad-CAM analysis demonstrated the most valuable information deeply mined by the DL signature in GR prediction, in which the weight distribution of the pixels was visualized by different colors, revealing that patients in the GR group commonly had larger red areas in their tumors. The distribution of DLCS scores in the TC and the images of Grad-CAM heatmaps of four tumors for different TRGs are shown in Figure 4. Figure S5, Supplemental Digital Content 2, http://links.lww.com/JS9/A434 displays two reconstructed ROIs from randomly selected GR samples during training.

### Survival model performance

To further evaluate the survival benefits of the proposed signature, we collected the follow-up data of 654 LAGC patients. A total of 265 patients died within 3 years after therapy, and the 3-year OS was 59.48%. The detailed characteristics of the enrolled patients are shown in Table S1, Supplemental Digital Content 2, http://links.lww.com/JS9/A434. From the results of univariable and multivariable Cox regression analyses (Table 3), we observed that the employed DL signature was an independent risk factor associated with OS [hazard ratio (HR), 0.827, 95% CI, 0.730–0.941, log-rank test, *P*=0.004].

**Table 3:** Univariate and multivariate analysis of predictors of OS

	Univariate			Multivariate		
Parameters	HR	95% CI	*P*	HR	95% CI	*P*
Age	0.989	0.977–1.001	0.085			
Sex	0.866	0.662–1.132	0.292			
BMI	1.014	0.972–1.058	0.508			
cT stage	0.276	0.830–2.430	0.422			
cN stage	0.612	0.783–1.466	<0.001^*^	1.052	0.892–1.240	0.548
cM stage	1.523	1.124–2.062	0.007^*^	1.031	0.918–1.158	0.605
Number of enlarged lymph nodes	1.072	1.031–1.115	<0.001^*^	1.088	0.933–1.268	0.284
Maximum diameter	1.042	1.017–1.066	0.001^*^	1.132	1.001–1.279	0.048
Thickness of gastric wall	1.088	0.951–1.246	0.221			
Tumor location						
Upper 1/3	Ref			Ref		
Middle1/3	1.276	0.974–1.670	0.077			
Lower 1/3	0.820	0.635–1.059	0.128			
Whole stomach	1.555	1.022–2.366	0.039^*^	1.037	0.924–1.163	0.540
Borrmann type						
I	Ref			Ref		
II	0.801	0.628–1.022	0.075			
III	0.971	0.749–1.258	0.822			
IV	1.670	1.257–2.22–	<0.001^*^	1.113	0.990–1.251	0.073
Pathological type	0.685	0.356–3.470	0.101			
Adenocarcinoma	Ref			Ref		
Mucinous adenocarcinoma	1.072	0.343–3.345	0.905			
Signet ring cell carcinoma	1.975	1.208–3.229	0.007^*^	1.019	0.906–1.145	
Squamous carcinoma	0.968	0.241–3.891	0.964			
Others	1.379	0.442–4.305	0.580			
SRC composition	1.618	1.221–2.143	0.001^*^	1.066	0.943–1.206	0.308
Differentiated degree	0.371	0.242–0.569	<0.001^*^	0.795	0.691–0.915	0.001^*^
CEA	1.500	1.171–1.921	0.001^*^	1.165	1.035–1.311	0.011^*^
CA125	1.637	1.191–2.251	0.002^*^	1.102	0.983–1.236	0.096
AFP	0.644	0.394–1.052	0.079			
Neut	0.987	0.926–1.053	0.693			
Lymph%	1.003	0.989–1.017	0.697			
PCT	0.199	0.042–0.932	0.040^*^	0.896	0.792–1.012	0.078
ALB	1.018	0.991–1.047	0.197			
Glu	0.937	0.848–1.035	0.199			
DL signature	0.369	0.221–0.617	<0.001^*^	0.828	0.730–0.941	0.004^*^

* *P*<0.05.

AFP, alpha-fetoprotein; ALB, albumin; BMI, body mass index; CA125, cancer antigen 125; CEA, carcinoembryonic antigen; cM, clinical metastasis; cN, clinical nodal; cT, clinical tumor; DL signature, deep learning signature; Glu, glucose; HR, hazard ratio; OS, overall survival; Neut, neutrophil level; PCT, platelet crit; Ref, reference; SRC, signet ring cell.

**Figure 5:**
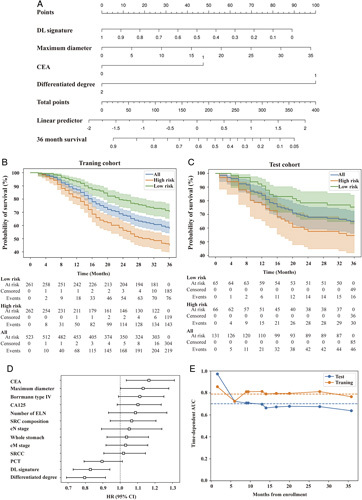
The relationship between DL signature and prognosis on the follow-up LAGC cohort. (A) The significant DL signature, maximum diameter of tumor, CEA level, and differentiated degree were the risk factors in LAGC. (B, C) Survival difference between high-risk group and low-risk group in the training cohort and test cohort. (D) Forest plot illustrating multivariable Cox regression analyses for prognosis in the follow-up cohort. (SE) Time-dependent AUC scores in the training cohort and test cohort. AFP, alpha-fetoprotein; AUC, area under the ROC curve; CEA, carcinoembryonic antigen; CA125, cancer antigen 125; cM, clinical metastasis; cN, clinical nodal; DL, deep learning; ELN, extra-lymph node metastases; LAGC, locally advanced gastric cancer; PCT, platelet crit.

In addition, 12 clinicopathologic characteristics were found to be associated with OS, including cN stage, cM stage, number of enlarged lymph nodes, maximum diameter, whole stomach, Borrmann type IV, SRC composition, differentiation degree, CEA level, CA125 level, and PCT level (Table 3, Fig. 5D). In the MCR analysis, the DL signature, differentiation degree and CEA level were identified as significantly independent risk factors for OS modeling (Fig. 5A). The threshold of the OS model was 0.0094, which was used to divide all experimental patients into two groups (high-risk and low-risk subsets). Kaplan–Meier analyses showed that the OS model could be used as a significant factor for the risk identification of OS (Fig. 5B, C). The values of the C-index, iAUC and IBS of the prognostic nomogram were 0.67, 0.79 and 1.24 in the training set and 0.64, 0.71 and 2.03 in the test set. The smoothed calibration curves of the OS models at 12, 24 and 35 months are provided in Figure S6, Supplemental Digital Content 2, http://links.lww.com/JS9/A434. The time-dependent ROC curves for the two GC datasets are shown in Figure 5E.

## Discussion

NCT is an important treatment for LAGC, but different patients have different responses to NCT. At present, there is no reliable and effective method for predicting the efficacy of NCT for LAGC, which leads to the failure of NCT in some patients with LAGC, and some patients even miss the chance of radical surgery due to disease progression during chemotherapy. Therefore, the development of an accurate predictive model to assess the efficacy of NCT prior to treatment is of great significance for the precise treatment of LAGC patients. In the present study, we proposed and validated an effective DL signature based on pretherapy CT images for TRG discrimination in LAGC patients treated with NCT. Furthermore, we verified the added value of the identified clinicopathological characteristics for predicting TRG and OS using the MLR and MCR methods.

In recent years, there has been growing interest in radiomics research due to its ability to extract and analyze a large number of advanced quantitative imaging features that may reflect the heterogeneity of the tumor. Radiomic features have demonstrated clinical value in the early prediction and identification of patients who may be sensitive to NCT. An earlier study found that the radiomics features screened by CT imaging before treatment are important markers of the response to NCT in LAGC^28^. Sun *et al*.^29^ performed radiomic feature extraction on the portal vein CT images of 106 GC patients before NCT and established an efficacy prediction model of NCT using a random forest algorithm, which showed perfect predictive performance in the validation cohort, with an AUC of 0.82. Zhou *et al*.^10^ extracted radiomic features from the CT images of 323 GC patients and found that the radiomics signature had good discrimination performance for predicting the NCT response in the external cohort (AUC, 0.679; 95% CI, 0.554–0.803). In addition, a radiomic model for predicting the efficacy of NCT in GC was constructed using a Bayesian classifier, support vector machine, random forest and other algorithms, and good discrimination performance was observed in both the IVC (AUC, 0.784; 95% CI, 0.659–0.908) and EVC (AUC, 0.803; 95% CI, 0.717–0.888)^30^. However, the clinical relevance of these findings is limited due to the relatively small sample sizes of the studies and the lack of validation in multicenter cohorts. Recently, the process of DL radiomic feature extraction was performed in a larger population (719 patients) for predicting the efficacy of NCT in GC, and higher AUCs of 0.804–0.829 were observed in the IVC and EVC. However, it lacked an end-to-end architecture for TRG prediction^31^. Therefore, we proposed an end-to-end DL signature to extract richer information from larger and more diverse datasets. The DLCS model in our study showed perfect performance in predicting the response to NCT in the IVC (AUC, 0.86) and EVC (AUC, 0.82), with good calibration in all cohorts (*P*>0.05).

The size of the GR group is typically several times larger than that of the PR group. Achieving a balanced imaging dataset can be resource-intensive or lack algorithmic complexity, which can result in unstable results, particularly when a large number of images need to be generated. Accordingly, we used a state-of-the-art oversampling algorithm, DeepSMOTE, to enrich the information in the GR group in an attempt to generate more GR images to improve the discriminative performance of the model. The visualized output map of the tumor area, which reveals the imaging characteristics extracted by the DL signature associated with intratumor heterogeneity, may provide valuable information for predicting TRG in GC. Analysis of the heatmaps revealed that the reconstructed tumors were able to capture most of the important features that were used for decision-making. Moreover, we explored the effectiveness of the DL signature in predicting OS. Our previous study found that the TRG score was related to LAGC patient prognosis after D2 gastrectomy^3^. In this study, we found that the DL signature was an independent risk factor for survival in LAGC patients treated with NCT. Patients with higher DL signature scores had better OS. More specifically, patients with GR after NCT could benefit greatly in terms of survival. Our study also identified several independent risk factors for survival in LAGC patients, including low differentiation, Borrmann type IV, high pre-NCT CEA levels, and cN stage. These findings are consistent with those of many other previous studies. Therefore, the proposed nomogram may provide a feasible way to guide treatment plans and implement personalized treatment for LAGC patients treated with NCT.

However, this study has several limitations. Firstly, as a retrospective and multicenter study, there may be potential selection bias and inherent bias. For example, patients from different levels of hospitals using different CT devices may cause bias. Therefore, in order to validate the generalizability and clinical applicability of our models, it is necessary to design prospective studies. Secondly, although we visualized the intratumor characteristics extracted by the DL signature, its clear biological significance is still unknown and needs to be fully elucidated. Further exploration of the relationship between radiographic features and the tumor microenvironment may provide additional microlevel information and elucidate the biological significance of the DL signature. Thirdly, the data on the DFS in this study was lacking. Fourthly, the imaging features extracted are largely dependent on the ROIs. However, the precise manual delineation of tumor margins requires professional expertise and is highly influenced by subjective experience. Therefore, an automated tumor segmentation mechanism for CT images in GC needs to be further developed for more precise TRG prediction. Fifthly, validation of the clinical reliability of the images generated by DeepSMOTE in the GR group should be explored in future studies since the algorithm has only been widely assessed on natural images. Sixthly, a one-stage network design should be further developed to decrease the information loss which may be caused by the disentanglement methods in modeling.

## Conclusion

In conclusion, we developed and validated a model that combines DL signature and clinical factors, which has demonstrated promising performance in predicting the response to NCT and prognosis in LAGC patients. Our model provides valuable information for guiding treatment plans and implementing personalized treatment strategies for LAGC patients receiving NCT. However, prospective studies are needed to validate the generalizability and clinical applicability of our DLCS model.

## Ethical approval and consent to participate

This study was approved by the ethics committee of each participating hospital. The requirement for informed consent was waived. The authors are accountable for all aspects of the work in ensuring that questions related to the accuracy or integrity of any part of the work are appropriately investigated and resolved.

## Consent for publication

Not applicable.

## Sources of funding

This study was supported by the National Key Research and Development Program of China (2021YFA0910100), Zhejiang Provincial Research Center for Upper Gastrointestinal Tract Cancer (JBZX-202006), Medical Science and Technology Project of Zhejiang Province (WKJ-ZJ-2202, WKJ-ZJ-2104), National Natural Science Foundation of China (82074245, 81973634, 81903842), Natural Science Foundation of Zhejiang Province (LR21H280001), Science and Technology Projects of Zhejiang Province (2019C03049), and Program of Zhejiang Provincial TCM Sci-tech Plan (2018ZY006, 2020ZZ005).

## Author contribution

Z.X., Y.D., and X.C.: conception and design; X.C.: administrative support; C.H. and W.C.: provision of study materials or patients; C.H. and F.L.: data analysis and interpretation; F.L.: deep learning methods; C.H., F.L., and Z.X.: manuscript writing. All authors contributed to the collection and assembly of data and were also involved in the final approval of the manuscript.

## Conflicts of interest disclosure

The authors declare that there are no conflicts of interest.

## Data availability statement

Due to the privacy of patients, the data related to patients cannot be available for public access but can be obtained from the corresponding author on reasonable request approved by the institutional review board of all enrolled centers.

## Provenance and peer review

Not commissioned, externally peer-reviewed.

## Supplementary Material
