# Supplementary material for: Deep learning radio-clinical signatures for predicting neoadjuvant chemotherapy response and prognosis from pretreatment CT images of locally advanced gastric cancer patients
Source: Int J Surg. 2023 May 3;109(7):1980–92. doi: 10.1097/JS9.0000000000000432 (PMC10389454; doi:10.1097/JS9.0000000000000432)
Supplement: Supplementary file 2 [file js9-109-1980-s002.docx]

**Supplemental material**

1. **Table S**

**Table S1 Clinicopathological characteristics of LAGC patients in the training and validation cohorts in the follow-up cohort.**

| **Characteristics** | **Training cohort**  **（523）** | **Test cohort**  **（131）** | **P value** |
| --- | --- | --- | --- |
| **Age (year)** |  |  |  |
| **≥60** | 286 | 78 | 0.317 |
| **＜60** | 237 | 53 |  |
| **Sex** |  |  |  |
| **Female** | 131 | 38 | 0.355 |
| **Male** | 392 | 93 |  |
| **BMI (Kg/m^2)** |  |  |  |
| **＜18.5** | 48 | 12 | 0.818 |
| **18.5-23.9** | 336 | 93 |  |
| **≥24.0** | 109 | 26 |  |
| **Location** |  |  |  |
| **Upper 1/3** | 164 | 47 | 0.653 |
| **Middle1/3** | 128 | 29 |  |
| **Lower 1/3** | 195 | 44 |  |
| **Whole stomach** | 36 | 11 |  |
| **Maximum diameter** |  |  |  |
| **＞5 cm** | 382 | 97 | 0.816 |
| **≤5 cm** | 141 | 34 |  |
| **Borrmann type** |  |  |  |
| **I+II** | 256 | 73 | 0.165 |
| **III+IV** | 267 | 58 |  |
| **cT stage** |  |  |  |
| **T1+T2** | 41 | 6 | 0.196 |
| **T3+T4** | 482 | 125 |  |
| **cN stage** |  |  |  |
| **N0+N1** | 119 | 32 | 0.684 |
| **N2+N3** | 404 | 99 |  |
| **cM stage** |  |  |  |
| **M0** | 443 | 113 | 0.655 |
| **M1** | 80 | 18 |  |
| **Differentiated degree** |  |  |  |
| **Poorly/Poorly-Middle** | 431 | 109 | 0.830 |
| **Middle/Well** | 92 | 22 |  |
| **Pathological type** |  |  |  |
| **Adenocarcinoma** | 486 | 122 | 0.935 |
| **others** | 37 | 9 |  |
| **CEA** |  |  |  |
| **Positive** | 168 | 41 | 0.856 |
| **Negative** | 355 | 90 |  |
| **CA125** |  |  |  |
| **Positive** | 72 | 14 | 0.351 |
| **Negative** | 451 | 117 |  |
| **AFP** |  |  |  |
| **Positive** | 50 | 8 | 0.214 |
| **Negative** | 473 | 123 |  |
| **ALB** | 40.04±4.47 | 40.04±3.902 | 0.694 |
| **PCT** | 0.26±0.08 | 0.25±0.08 | 0.757 |
| **Lymph%** | 1.60±0.08 | 1.71±0.67 | 0.049 |
| **Glu** | 5.69±1.50 | 5.59±1.13 | 0.487 |
| **Neut** | 4.13±1.93 | 4.00±2.11 | 0.514 |

**2 Figure S**


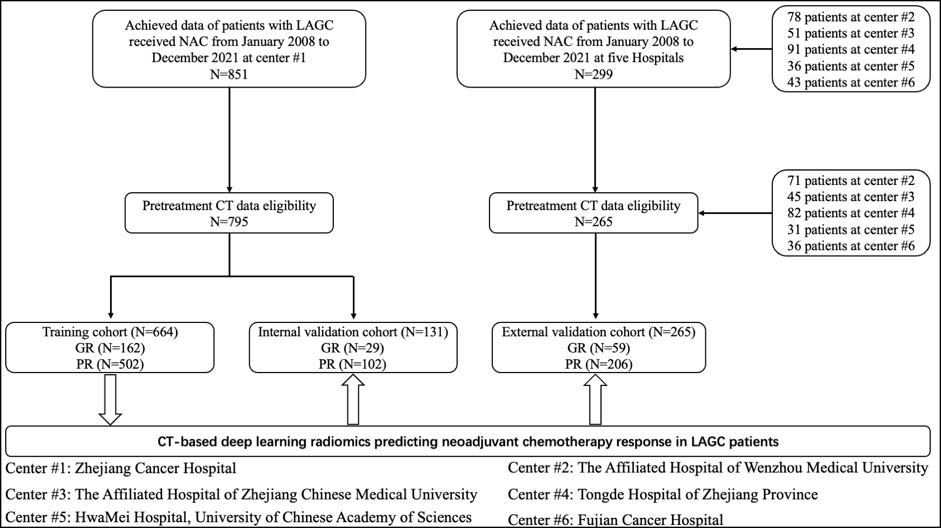


**Figure S1: Flowchart Illustrating the Study Recruitment.**

**
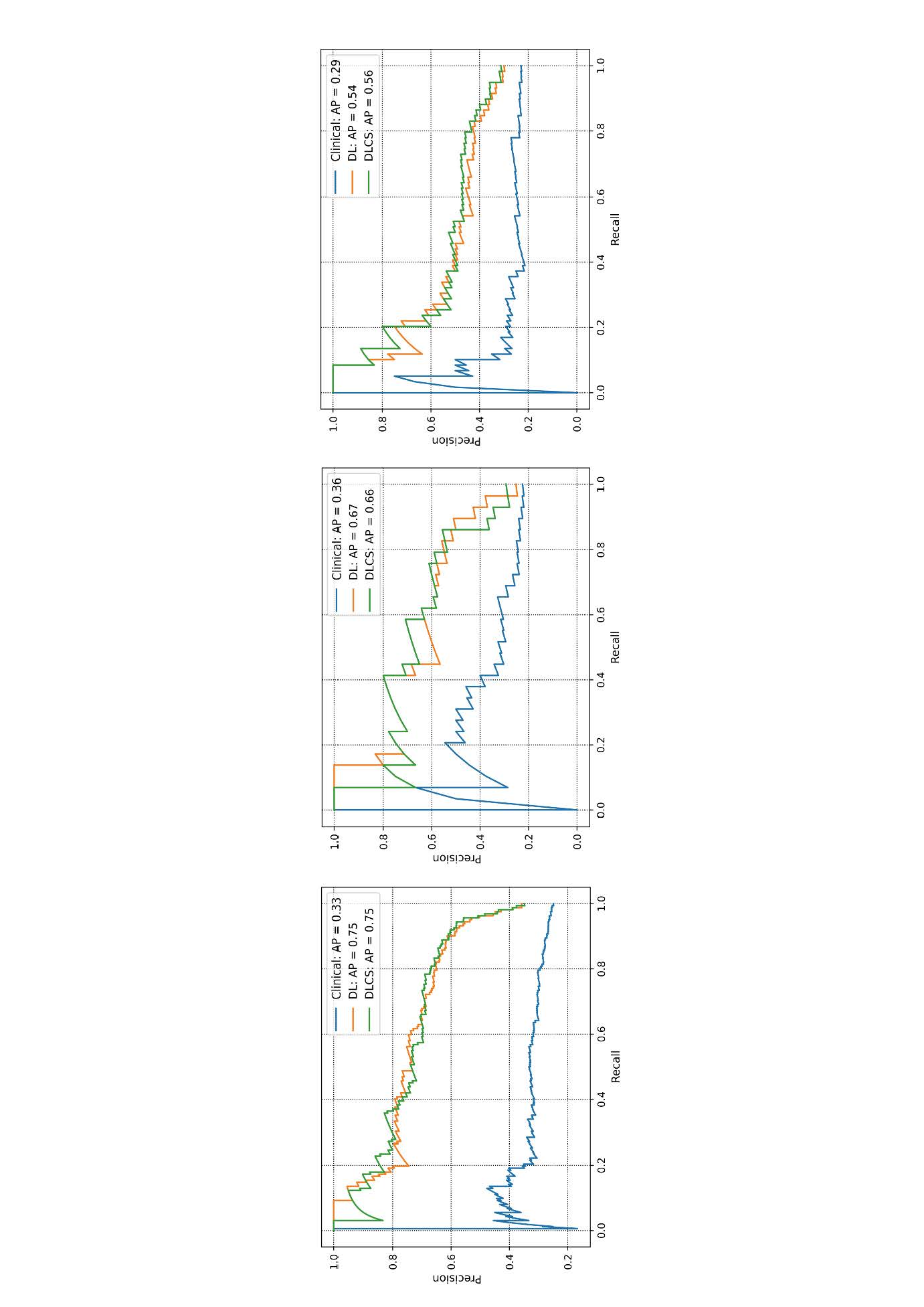
**

**Figure S2: The PR curves of all models. A: Training cohort. B: Internal validation cohort. C: External validation cohort.**

**
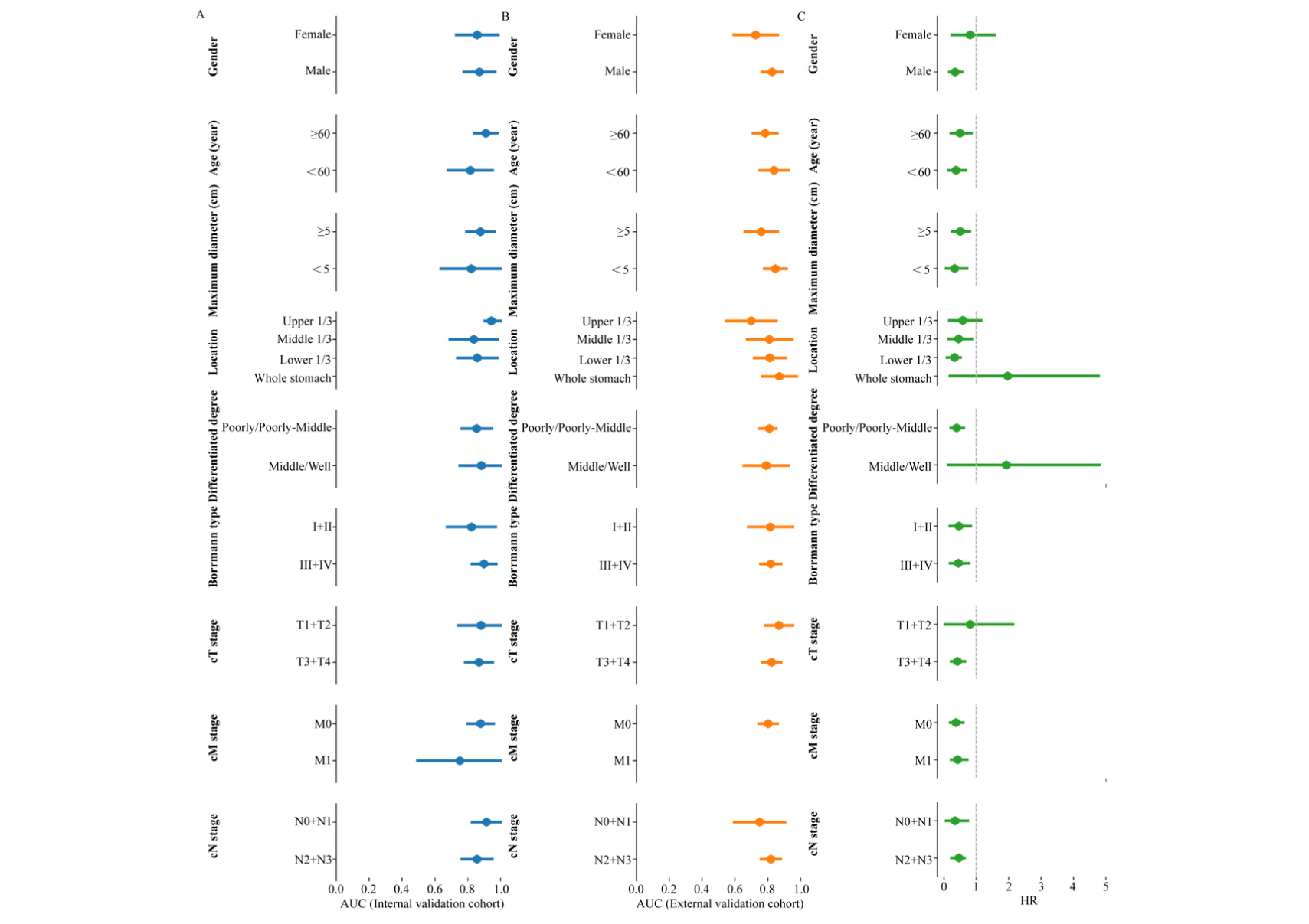
**

**Figure S3: Predictive value of DL signature for chemotherapy response and survival in subgroups. A: Internal validation cohort. B: External validation cohort. C: The hazard ratio in survival analysis.**

**
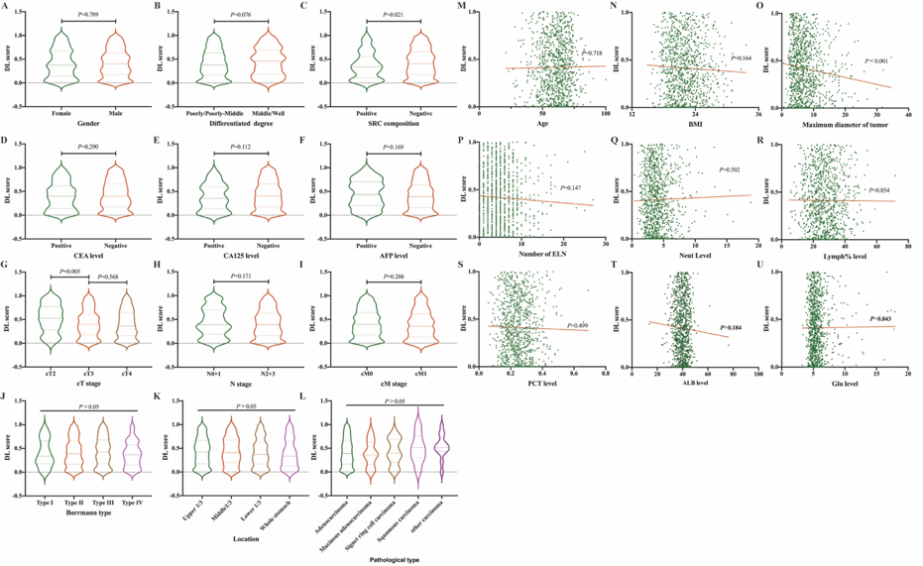
**

**Figure S4: The relationship between DL score and clinicopathologic characteristics**


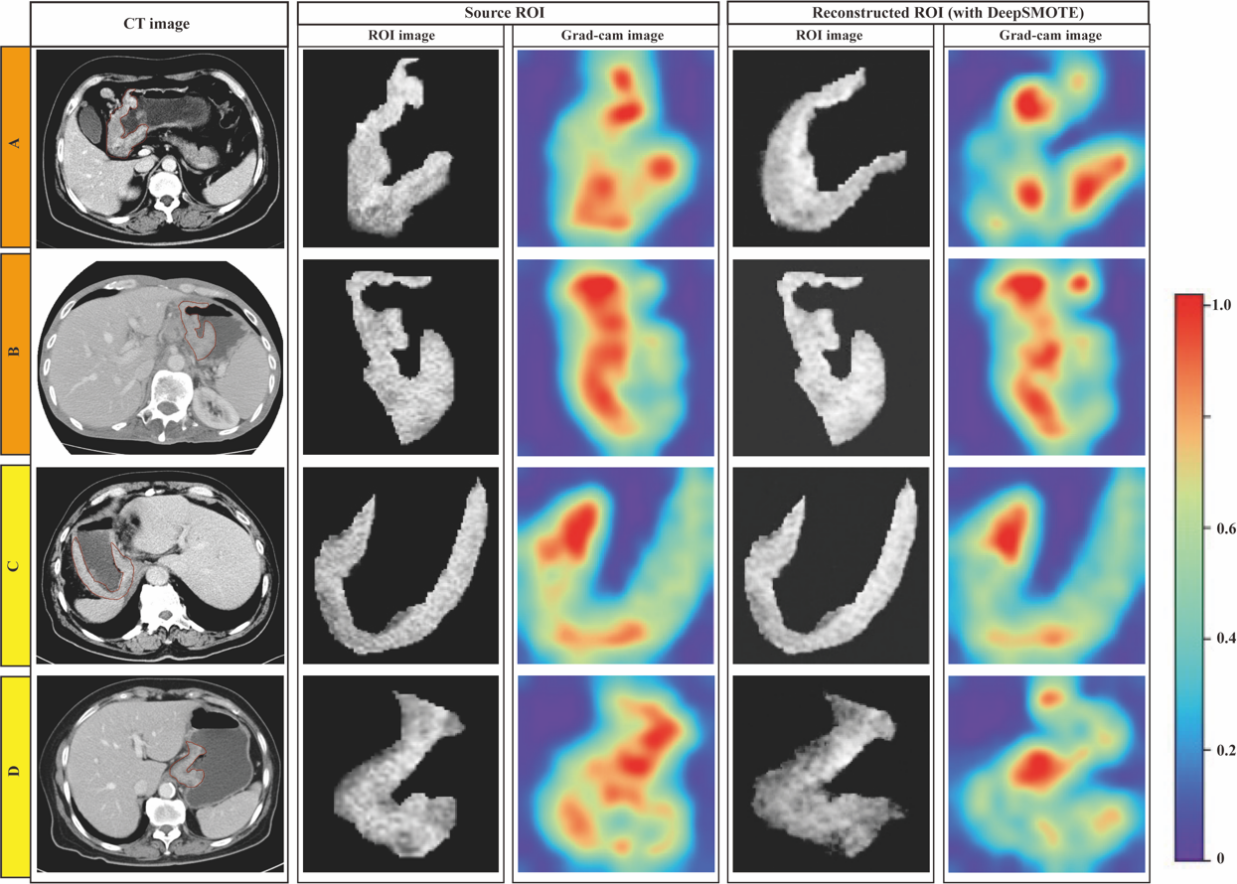


**Figure S5: The reconstructed ROI with DeepSMOTE was compared to the original ROI from randomly selected GR samples during training.**


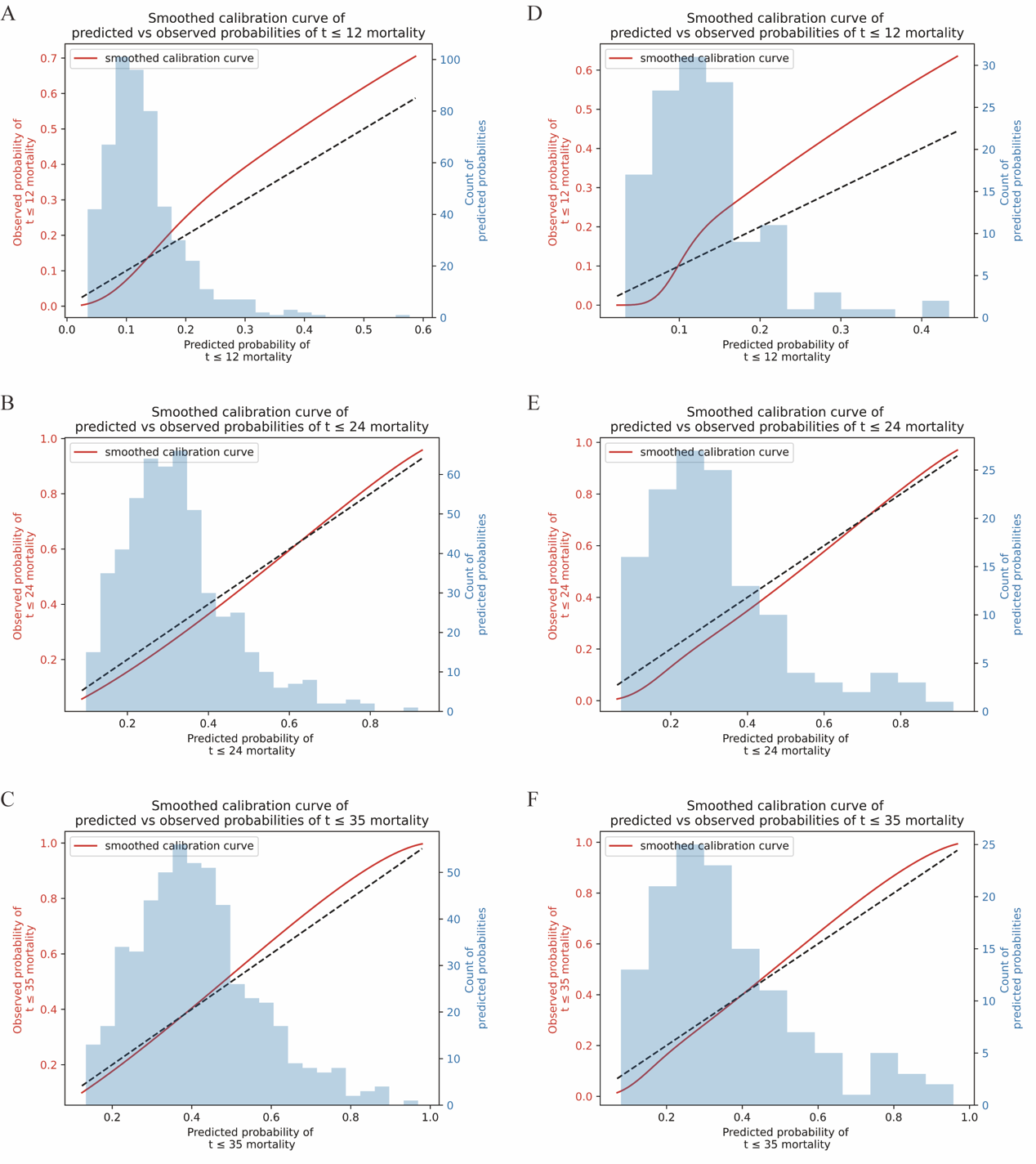


**Figure S6: The smoothed calibration curves of OS models in 12, 24 and 35 months**

**3 Function S**

1. **SE block function**

$$z_{c}=\boldsymbol{F}_{sq}(\boldsymbol{u}_{c})=\frac{1}{W\times H}\sum_{i=1}^{W} \sum_{j=1}^{H} u_{c}(i,j), c\epsilon\{1,C\}$$

$$\boldsymbol{s}=\boldsymbol{F}_{ex}(\mathbf{z},\boldsymbol{W})=\sigma(g(\mathbf{z},\boldsymbol{W}))=\sigma(\boldsymbol{W}_{2}\delta(\boldsymbol{W}_{1}\mathbf{z}))$$

$$\tilde{x}_{c}=\boldsymbol{F}_{scale}(\boldsymbol{u}_{c},s_{c})=s_{c}\cdot\boldsymbol{u}_{c}$$

Where **U** is the feature map with the size of (**C**, **W**, **H**); $\boldsymbol{W}_{1}\epsilon{\mathbb{\mathbb{R}}}^{\frac{C}{r}\times C},\boldsymbol{W}_{2}\epsilon{\mathbb{\mathbb{R}}}^{C\times\frac{C}{r}}$ are the weight matrices for the two fully connected layers in SE block respectively, $\delta$ and $\sigma$represent the ReLU and sigmoid activation functions respectively,$\boldsymbol{s}$ is called as gate mechanism; $\tilde{x}_{c}$ is one of feature maps for the output $\tilde{\mathbf{X}}$.
